# 2D-HSQC-NMR-Based Screening of Feruloylated Side-Chains of Cereal Grain Arabinoxylans

**DOI:** 10.3389/fpls.2022.951705

**Published:** 2022-07-07

**Authors:** Rachel R. Schendel, Mirko Bunzel

**Affiliations:** ^1^Department of Animal and Food Sciences, University of Kentucky, Lexington, KY, United States; ^2^Department of Food Chemistry and Phytochemistry, Institute of Applied Biosciences, Karlsruhe Institute of Technology (KIT), Karlsruhe, Germany

**Keywords:** arabinoxylan, ferulic acid, 2D-NMR, HSQC, feruloylated side-chains

## Abstract

Arabinoxylans of commelinid monocots are characterized by high contents of ferulic acid that is incorporated into arabinose-bearing side-chains of varying complexity. Species-related differences in the feruloylated side-chain profiles of grain arabinoxylans are observed and lead to differences in arabinoxylan functionality. Here, a semi-quantitative assay based on ^1^H-^13^C-correlation NMR spectroscopy (HSQC experiment) was developed to profile feruloylated side-chains of cereal grain arabinoxylans. Following acidic liberation of the feruloylated side-chains from the xylan backbone and a clean-up step using C18 solid phase extraction, the feruloylated oligosaccharides FA (5-*O*-*trans*-feruloyl-L-arabinofuranose), FAX (β-d-xylopyranosyl-(1 → 2)-5-*O*-*trans*-feruloyl-l-arabinofuranose) and FAXG (α-l-galactopyranosyl-(1 → 2)-β-d-xylopyranosyl-(1 → 2)-5-*O*-*trans*-feruloyl-l-arabinofuranose) were analyzed by HSQC-NMR. Marker signals were identified for each compound, and experimental conditions such as solvent and internal standard as well as measurement and processing conditions were optimized for a semi-quantitative determination. The approach was validated with respect to accuracy, precision, limit of detection, and limit of quantification. The newly developed approach was applied to several cereal samples including oats, popcorn maize, wheat, and wild rice. Data were compared to an HPLC-DAD/MS approach published earlier by our group, demonstrating that the results of the HSQC approach were comparable to the more time-consuming and technically more challenging HPLC-DAD/MS method.

## Introduction

**Figure 1:**
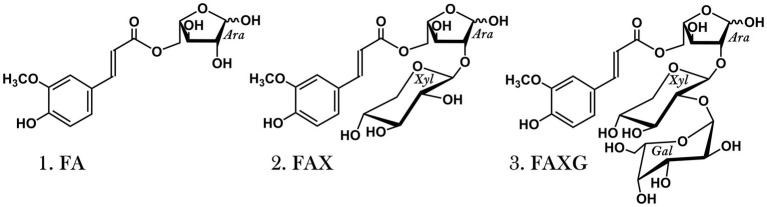
Feruloylated side chain standard compounds released from maize arabinoxylan *via* mildly acidic hydrolysis, purified, and used for method development. 1. FA: 5-*O*-*trans*-feruloyl-l-arabinofuranose. 2. FAX: β-d-xylopyranosyl-(1 → 2)-5-*O*-*trans*-feruloyl-l-arabinofuranose. 3. FAXG: α-l-galactopyranosyl-(1 → 2)-β-d-xylopyranosyl-(1 → 2)-5-*O*-*trans*-feruloyl-l-arabinofuranose.

Arabinoxylans (AX) are the dominant hemicelluloses in commelinid monocots, including cereal grains (Poaceae; Scheller and Ulvskov, 2010). The xylan backbones of these polysaccharides are substituted with l-arabinofuranose and l-arabinofuranose-containing oligosaccharides, and some substituents are acylated with ferulic acid, forming feruloylated side-chains of varying complexity (Saulnier et al., 1995; Allerdings et al., 2006). Other hydroxycinnamic acids, such as *p-*coumaric acid, are also associated with AX (Mueller-Harvey et al., 1986; Ishii, 1997), but in the grain tissue of monocots, the ferulates are far more concentrated than any other hydroxycinnamate (Vitaglione et al., 2008). Free-radical-induced oxidative coupling of the monomeric ferulates produces ferulate dimers and higher oligomers, which stabilize cereal grains’ cell walls by covalently cross-linking AX to each other and lignin (Ralph et al., 1994, 1995; Allerdings et al., 2005; Bunzel, 2010). However, many of the ferulates remain in their original monomeric form (Bunzel, 2001), and key characteristics of AX, particularly those related to enzymatic digestibility and microbial fermentation, are mediated not only by ferulate dimers’/higher oligomers’ crosslinking of cell wall polymers, but also by the abundance and structural complexity of their monomeric feruloylated side-chain substituents. For example, many arabinofuranosidases cannot cleave even the most simple feruloylated side-chain, feruloylated arabinose (FA; 5-*O*-*trans*-feruloyl-l-arabinofuranose; see Figure 1), from the xylan backbone (Wood and McCrae, 1996; Luonteri et al., 1999; Rémond et al., 2008; Schendel et al., 2016a). A study focused on the maize grain fraction resistant to mild acid pretreatment and subsequent enzymatic saccharification found that feruloylated oligosaccharides made up 39% of the enzyme-resistant oligosaccharides, and most of these feruloylated oligosaccharides contained either the complex, feruloylated trisaccharide side-chain FAXG (α-l-galactopyranosyl-(1 → 2)-β-d-xylopyranosyl-(1 → 2)-5-*O*-*trans*-feruloyl-l-arabinofuranose; see Figure 1) or the feruloylated disaccharide FAX (β-d-xylopyranosyl-(1 → 2)-5-*O*-*trans*-feruloyl-l-arabinofuranose; see Figure 1) side-chains (Appeldoorn et al., 2013). Because more complex AX structures contain additional glycosidic linkage types compared to simple AX (Feijao et al., 2022), more elaborate enzymatic machinery is needed to break down these complex AX (Rogowski et al., 2015; Beri et al., 2020). Unique microbial growth and fermentation patterns thus arise from different AX based on which microbial population is best equipped to compete for energy from the AX food source, i.e., produce the enzymes needed to break down the polymer into fermentable monosaccharides (Yang et al., 2014; Centanni et al., 2017; Mendis et al., 2018; Ndeh and Gilbert, 2018).

The ability to rapidly and quantitatively screen cereal grain materials for their feruloylated side-chain profiles will be helpful for unraveling the relationships between diet composition, carbohydrate structure, and gut microbial population shifts. We previously developed and validated an HPLC-DAD/MS-based side-chain profiling method which allowed us to demonstrate clear species-related differences in the feruloylated side-chain profiles of 12 different grain AX (Schendel et al., 2016b). This approach utilized a mildly acidic hydrolysis (50 mM TFA, 1 h) to partially release the feruloylated side-chains. On-column mutarotation between the α- and β-anomers of the released side-chains’ reducing ends resulted in broad, tailing, split HPLC peaks and necessitated a reduction step to transform the side-chain compounds to sugar alcohols before HPLC analysis. Lapierre et al. (2018) published an alternative method utilizing acidolysis in dioxane/methanol, which converts the side-chain compounds to methyl glycosides and eliminates the need for reduction. However, both methods require chromatographic separation of hydrolysis mixtures, and we have therefore developed a 2D-NMR-based method which eliminates the need for chromatography and streamlines AX side-chain profiling of cereal grain materials.

## Materials and Methods

### Plant Materials and Isolated Standard Compounds

Wholegrain popcorn (*Zea mays* L. var. *everta*), wild rice (*Zizania aquatica* L.), wheat (*Triticum aestivum* L.), and oats (*Avena sativa* L.; dehulled) were obtained from a local grocery store and milled (< 0.5 mm). *Insoluble fiber material* was isolated from the milled grains using α-amylase-based starch digestion followed by extensive washing and drying as described in Schendel et al. (2016b). All fiber samples were corrected for protein (Kjeldahl; N × 6.25) and ash (gravimetric detection after incineration). The *standard compounds FA, FAX, and FAXG* (Figure 1) were isolated and purified from maize middlings as described by Schendel et al. (2016b).

### Chemicals and Laboratory Supplies

Dimethyl sulfoxide-*d*_6_ (≥99.9%; DMSO), methanol (MeOH), and trifluoroacetic acid (TFA) were from Sigma (Seelze, Germany). Caffeine was purchased from Carl Roth (Karlsruhe, Germany). Water was deionized and filtered with a Milli-Q Reference water purification system (Merck Millipore, Billerica, Massachusetts, United States). Solid-phase extraction (SPE) cartridges (Chromabond C18, 500 mg bed weight, 3 ml cartridge volume) were from Macherey-Nagel (Düren, Germany).

### Semi-Selective Release of Feruloylated Side-Chains *via* Acidic Hydrolysis and Clean-Up

Dry insoluble fiber (100 mg for corn fiber; 200 mg for other grain fibers; all samples analyzed in triplicate) was weighed into a 15-mL Pyrex tube, capped tightly, and hydrolyzed in the dark (5 ml 50 mM TFA, 100°C, 2 h). Following hydrolysis, samples were cooled on ice and centrifuged. An aliquot of supernatant (2–3 ml, record exact volume) was applied to a preconditioned (6 ml MeOH, 6 ml H_2_O) SPE cartridge. Loaded cartridges were washed dropwise with water (6 ml), and feruloylated side-chains were eluted with MeOH (6 ml). Eluted samples were evaporated to dryness (rotary evaporation and vacuum drying oven).

### NMR Method Development

All NMR analyses were performed on a Bruker (Ettlingen, Germany) Avance 500 MHz system set up with a Prodigy CryoProbe. HSQC spectra were obtained with the standard Bruker pulse sequence HSQCETGP and the following parameters: relaxation delay: 1.5 s; ^1^*J*_CH_ coupling constant: 145 Hz; 256 and 1,024 points recorded in the f1 and f2 dimensions, respectively. 20 scans were acquired per sample. Data were processed using a squared sine window function (QSINE) in both dimensions. Spectra were calibrated against residual DMSO using the chemical shifts (^1^H = 2.50 ppm; ^13^C = 39.52 ppm) published by Gottlieb et al. (1997). The selected quantification signals for FA, FAX, and FAXG (see “Results and Discussion”) were volume-integrated. Caffeine’s most downfield methyl cross-peak signal (in DMSO, ^1^H: 3.88 ppm; ^13^C: 33.11 ppm) was used as the internal standard signal and also volume-integrated. The ratios of the feruloylated standard compounds’ signal volumes to the caffeine signal volume were determined and used for calibration and quantification.

Five-point equidistant internal standard curves in DMSO-*d*_6_ were prepared in triplicate for each of the three feruloylated standard compounds [FA (470–2,400 μM), FAX (120–440 μM), and FAXG (120–350 μM)] using caffeine as the internal standard (4,407 μM). Linear standard curve equations were generated using ordinary least-squares regression in OriginPro.

The limit of detection (LOD) and limit of quantification (LOQ) were calculated from each compound’s calibration data set using the following equations (Eqs. (1) and (2)) from the International Council for Harmonisation of Technical Requirements for Pharmaceuticals for Human Use guidelines (Ermer and Miller, 2006), where *SD_intercept_* is the standard deviation of the intercept and *b* is the slope of the regression line:


(1) $$LOD=\frac{3.3\;\left(SD_{intercept}\right)}{b}$$

(2) $$LOQ=\frac{10\;\left(SD_{intercept}\right)}{b}$$

Recovery of FA, FAX, and FAXG was determined in duplicate for each compound by subjecting freshly-prepared aqueous aliquots of known concentration to the SPE clean-up steps described in section “Semi-Selective Release of Feruloylated Side-Chains Via Acidic Hydrolysis and Clean-Up”. After drying, the residues were dissolved in 550 μl of internal standard solution (4,407 μM caffeine in DMSO-*d*_6_), transferred to a 5 mm diameter NMR tube, and measured using the HSQC-NMR parameters described in section “NMR Method Development”.

### Semi-Quantitative NMR Measurement of Feruloylated Side-Chains

Completed samples from section “Semi-Selective Release of Feruloylated Side-Chains Via Acidic Hydrolysis and Clean-Up” were dissolved in 550 μl of internal standard solution (4,407 μM caffeine in DMSO-*d*_6_) and transferred to a 5 mm diameter NMR tube. HSQC-NMR analyses were conducted using the same NMR instrument and parameters as described in section “NMR Method Development”. Spectra were calibrated against residual DMSO. Quantification signals were identified by overlaying spectra from standard compounds and volume-integrated.

### Quantification of Total Ester-Linked Ferulates in Cereal Fibers

The content of total ester-linked *trans*-ferulic acid was determined in the prepared insoluble cereal fibers (section “Plant Materials and Isolated Standard Compounds”) using the method described by Dobberstein and Bunzel (2010), with slight modifications. Dried insoluble fiber (25–50 mg for popcorn, wild rice, and wheat; 100 mg for oats; all samples analyzed in triplicate) was stirred with 5 ml of 2 M NaOH and the internal standard compound *ortho*-coumaric acid (50 μl of 5 mM *ortho-*coumaric acid in 50/50 MeOH/H_2_O, v/v) for 18 h in the dark at room temperature. Hydrolysates were acidified (0.95 ml 37% HCl), and the protonated phenolic acids were extracted into diethyl ether three times (6, 5, and 5 ml). The ether extracts were combined and dried under an N_2_ stream. The dried sample residues were dissolved in 1 ml of MeOH/H_2_O (50/50, v/v) and separated *via* HPLC on a Phenomenex Luna phenyl-hexyl column (250 × 4.6 mm, 5 μm particle size) using the following ternary, linear gradient: 1 mM TFA in water (eluent A); acetonitrile/1 mM TFA in water, 90/10, v/v (eluent B); and MeOH/1 mM TFA in water, 90/10, v/v (eluent C). Gradient steps were as follows: initial conditions, 87% A, 13% B; hold for 11 min; B from 13 to 15% in 12 min; B from 15 to 16% in 5 min; B from 16 to 50% and C from 0 to 25% in 4 min; B from 50 to 13%, and C from 25 to 0% in 1 min; followed by a re-equilibration step. The injection volume was 10 μl, and the flow rate was 1 ml/min. Compounds were detected at 325 nm in a diode array detector, and *trans-*ferulic acid was quantified with a linear, equidistant, 11-point internal calibration curve (5–2,500 μM), using *ortho*-coumaric acid as the internal standard (250 μM).

## Results and Discussion

NMR has been used to quantitatively rank different structural elements in AX. Many reports use ^1^H-NMR (Viëtor et al., 1994; Dervilly et al., 2002; Cyran et al., 2003; Feng et al., 2018), but signal overlap in the proton dimension presents a challenge for some cereal samples (Hoffmann et al., 1992). 2D-NMR techniques increase resolution by dispersing signals from the often-crowded proton dimension (f_2_) into a second dimension (f_1_). We chose to develop a semi-quantitative HSQC method based on the increased chemical shift dispersion of ^13^C-based heteronuclear methods. HSQC, which shows couplings between a ^1^H nucleus vs. the nucleus of the carbon (^13^C) to which the proton is directly bonded, has been used qualitatively for structural characterization for decades, but has more recently also gained interest for (semi)quantitative analyses (Fels and Bunzel, 2022). HSQC cross-peak intensities are influenced by several factors, including variations in ^13^C-^1^H coupling constants (^1^*J*_C,H_), differing polarization transfer efficiency, and relaxation time (T_1_ and T_2_) differences. However, internal calibration using authentic standard compounds corrects for these potential differences and permits semi-quantitative work.

### Method Development: Selection of NMR Solvent, Internal Standard, and Quantification Signals

Important method parameters were optimized, including NMR solvent, internal standard, quantification cross-peaks, and measurement time. We tested D_2_O for its suitability, but spontaneous cleavage of a portion of the ester linkages of the feruloylated side-chain compounds occurred in this solvent, ruling out its use. The onset of the degradation was fairly rapid (within a few hours of dissolving the dried standard compounds), and its extent increased as the compounds remained in solution. In contrast, standard compounds dissolved in DMSO-*d*_6_ remained stable in solution for weeks (the longest tested period was 4 weeks). Caffeine was chosen as the internal standard because it is a solid at room temperature, is soluble in DMSO, does not react with the feruloylated compounds, and importantly, has clear HSQC cross-peaks which do not overlap with the feruloylated side-chain standards.

Compound-specific cross-peak signals for integration and calibration of the individual feruloylated side-chain compounds were selected (Table 1), thus enabling simultaneous quantification in the same NMR tube. The cross-peaks for H2/C2 of the Ara*f* in FA were chosen because these signals were easily differentiated from cross-peaks from the more complex side-chains. In solution, these compounds are in equilibrium between their α- and β- anomers, and the chemical shifts of other carbons and protons in the molecule are affected by the anomeric position. As a result, two unique signals arise for this cross-peak which correspond to the α- and β-anomers of FA. The average ratio between the α- and β-anomers of FA was 76:24, but ranged from 73:27 up to 87:13. Because the ratio varies between samples, more accurate results will be obtained by summing the volume integrals of both anomers’ cross-peaks than by integrating only the cross-peak of the α-anomer.

**Table 1:** HSQC cross-peak signals selected for quantification.

Standard compound and selected cross-peak signal		Chemical shifts of quantification signals in DMSO-*d*_6_^a^			
		H (from α-anomer compound)	C (from α-anomer compound)	H (from β-anomer compound)	C (from β-anomer compound)
FA	Ara C2/H2	3.76	82.4	3.71	76.5
FAX	Ara C2/H2	3.93	89.3	3.85^b^	83.0^b^
FAXG	Gal C5/H5	3.90	71.0	4.02^b^	71.0^b^

*Ara, arabinose; FA, 5-O-trans-feruloyl-l-arabinofuranose; FAX, β-d-xylopyranosyl-(1 → 2)-5-O-trans-feruloyl-l-arabinofuranose; FAXG, α-l-galactopyranosyl-(1 → 2)-β-d-xylopyranosyl-(1 → 2)-5-O-trans-feruloyl-l-arabinofuranose; Gal, galactose; HSQC, heteronuclear single quantum coherence spectroscopy*.

a *Spectra were calibrated against residual DMSO signal (^1^H = 2.50 ppm; ^13^C = 39.52 ppm)*.

b *Calibration curves for FAX and FAXG were prepared using only the cross-peaks from the α-anomer compound*.

FAX was also quantified using the H2/C2 cross-peak of its Ara*f* moiety, which, compared to that of FA, is substantially downfield-shifted in the carbon dimension because of the β-(1 → 2)-linked xylose unit (Table 1). FAXG’s Ara*f* H2/C2 cross-peak directly overlaps with that of FAX, so integration of this signal represents the sum of FAX, FAXG, and likely, more complex side-chains that begin with the FAX moiety but contain additional sugar monomers, such as those described by Allerdings et al. (2006), or oligosaccharidic side chains which contain a ferulate dimer (dehydrodiferulate) in place of a monomer, such as those described by Bunzel et al. (2008). FAXG is specifically quantified *via* its H5/C5 cross-peak of α-galactose (Table 1), and subtracting this value from the sum value of FAX + FAXG obtains the concentration of FAX. FAX and FAXG could be easily quantified separately in mixtures of standard solutions *via* their respective Xyl*p* anomer signals. These signals are, however, impractical for use in plant hydrolysates, which always include some feruloylated (and acetylated) oligosaccharides containing fragments of the xylan backbone. The anomeric cross-peaks from these backbone Xyl*p* moieties overlap with the side-chain Xyl*p* cross-peaks.

Similarly to FA, the chemical shifts of the cross-peaks chosen as quantification signals for FAX and FAXG differ slightly depending on whether they belong to a molecule whose reducing Ara*f* is in the α- or β-anomeric configuration. However, the concentrations of FAX and FAXG are quite low in some plant materials, leading to detection of only the quantification signals corresponding to the more abundant α-anomer. We therefore chose to prepare calibration curves for FAX and FAXG using only the integrals from the α-anomer signals.

The number of measurement scans was another key method parameter choice. It was necessary to balance method sensitivity with a realistic total measurement time in order to keep the method feasible for routine-screening use. We used 20 scans, which equated to a measurement time of around 2.25 h while also permitting quantification of both FAX and FA in the hydrolysates produced from 200 mg or less of cereal fibers.

### Method Validation

**Figure 2:**
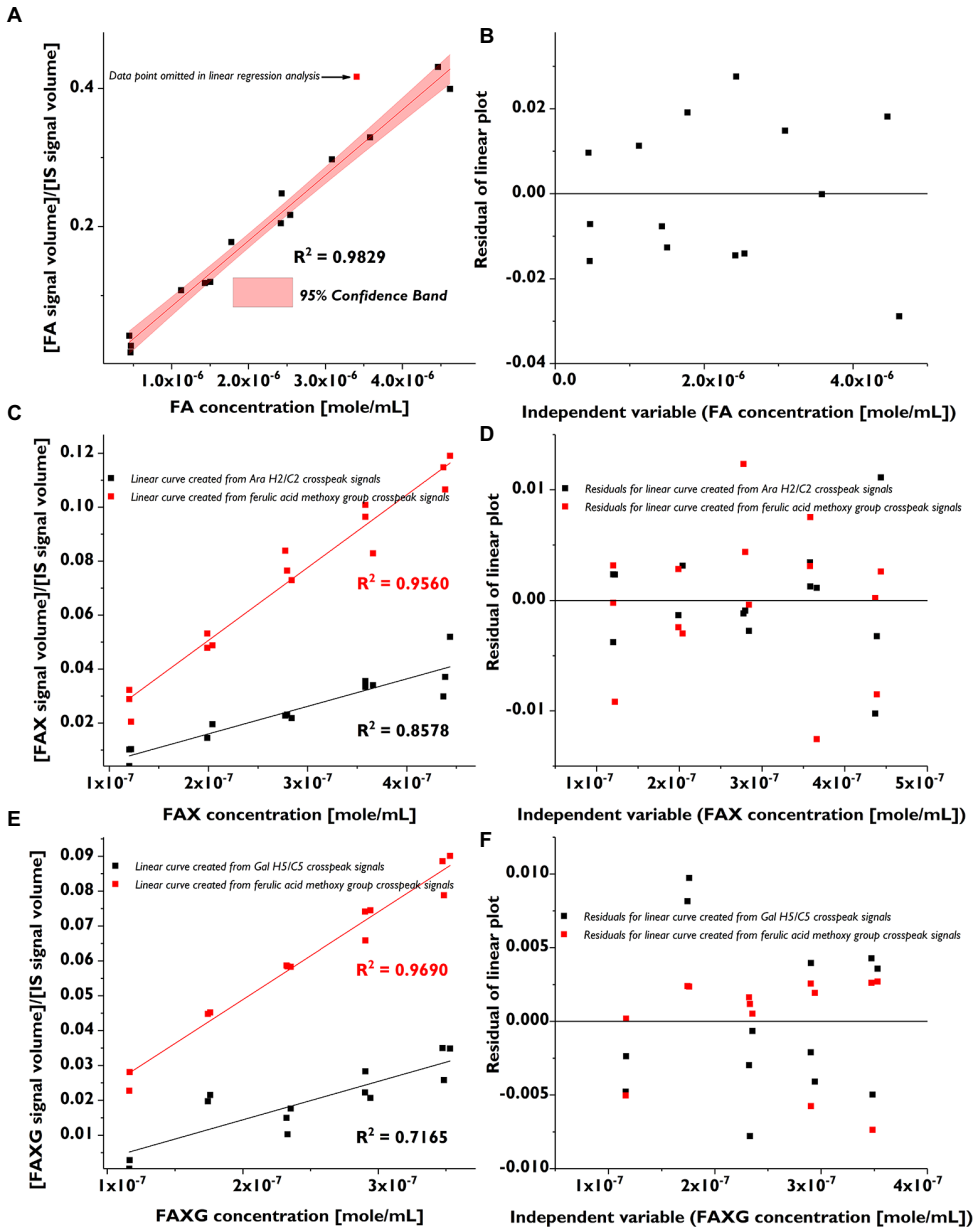
Linear calibration curves (in triplicate) and corresponding residual plots of feruloylated side-chain standard compounds following volume integration of selected HSQC cross-peaks. **(A)**: Linear calibration curve for FA. **(B)**: Residual plot for FA calibration curve. **(C)**: Linear calibration curve for FAX (black) created from the quantification cross-peak signal and the curve created from the ferulate methoxy cross-peak signal (red). **(D)**: Residual plots for FAX calibration curve (black) created from the quantification cross-peak signal and for the curve created from the ferulate methoxy cross-peak signal (red). **(E)**: Linear calibration curve for FAXG (black) created from the quantification cross-peak signal and the curve created from the ferulate methoxy cross-peak signals (red). **(F)**: Residual plots for FAXG calibration curve (black) created from the quantification cross-peak signal and for the curve created from the ferulate methoxy cross-peak signal (red). FA, 5-*O*-*trans*-feruloyl-l-arabinofuranose; FAX, β-d-xylopyranosyl-(1 → 2)-5-*O*-*trans*-feruloyl-l-arabinofuranose; FAXG, α-l-galactopyranosyl-(1 → 2)-β-d-xylopyranosyl-(1 → 2)-5-*O*-*trans*-feruloyl-l-arabinofuranose; HSQC, heteronuclear single quantum coherence spectroscopy. IS, internal standard.

We prepared low-range, linear calibration curves (in triplicate) for FA, FAX, and FAXG and used these to calculate the LOD and LOQ as described in section “NMR Method Development”. The calibration curves are shown in Figure 2, and their linearity was assessed by both visual inspection of the residual plots (Figure 2) and the correlation coefficients (Table 2).

**Table 2:** Calibration equations^a^, correlation coefficients, limits of detection (LOD), limits of quantification (LOQ), and recovery rates for FA, FAX, and FAXG using HSQC-NMR detection.

	Range tested (μM)	Linear calibration equation	Correlation coefficients (*R*^2^)	LOD (μM)	LOQ (μM)	Recovery rate (%)
FA	470–2,400	*y* = 99,366x – 0.0133	0.9829	307	930	103
FAX	120–440	*y* = 102,034x – 0.0044	0.8578	108	327	75
FAXG	120–350	*y* = 110,243x – 0.0076	0.7165	153	463	83

*FA, 5-O-trans-feruloyl-l-arabinofuranose; FAX, β-d-xylopyranosyl-(1 → 2)-5-O-trans-feruloyl-l-arabinofuranose; FAXG, α-l-galactopyranosyl-(1 → 2)-β-d-xylopyranosyl-(1 → 2)-5-O-trans-feruloyl-l-arabinofuranose; HSQC, heteronuclear single quantum coherence spectroscopy; NMR, nuclear magnetic resonance*.

a *Calibration equations and correlation coefficients calculated from triplicate calibrations. Recovery rates were determined in duplicate*.

The linearity of the FAX and FAXG calibration curves, prepared with only the volume integrals from the α-anomer, was weaker than that of the FA curve, which was prepared using the sum integral of the quantification cross-peaks from both the α- and β-anomers. The weaker linearity of the concentration: signal volume relationship for FAX and FAXG is due to the variability in the ratio of the α- and β-monomers in the compound solutions and not to sample handling errors. To demonstrate this, linear regression lines generated from the same FAX and FAXG data sets, but substituting the ferulate methoxyl cross-peak volume integrals, which are not affected by the ratio of α- and β- anomers, resulted in correlation coefficients >0.95 (Figure 2). Based on the limited linearity of the FAX and FAXG calibration curves prepared from the volume integral of only the α-anomer’s cross-peak, the FAX and FAXG sample concentrations must be treated as semi-quantitative results.

Method recovery rates for concentrations near the limit of quantification should lie between 70 and 120% (Vogelgesang and Hädrich, 1998). Good recovery rates (Table 2) for FA, FAX, and FAXG with the method were shown by subjecting standard solutions of FA, FAX, and FAXG to the sample preparation and analysis procedure beginning at SPE-clean-up.

### Method Application to Cereal Samples

**Figure 3:**
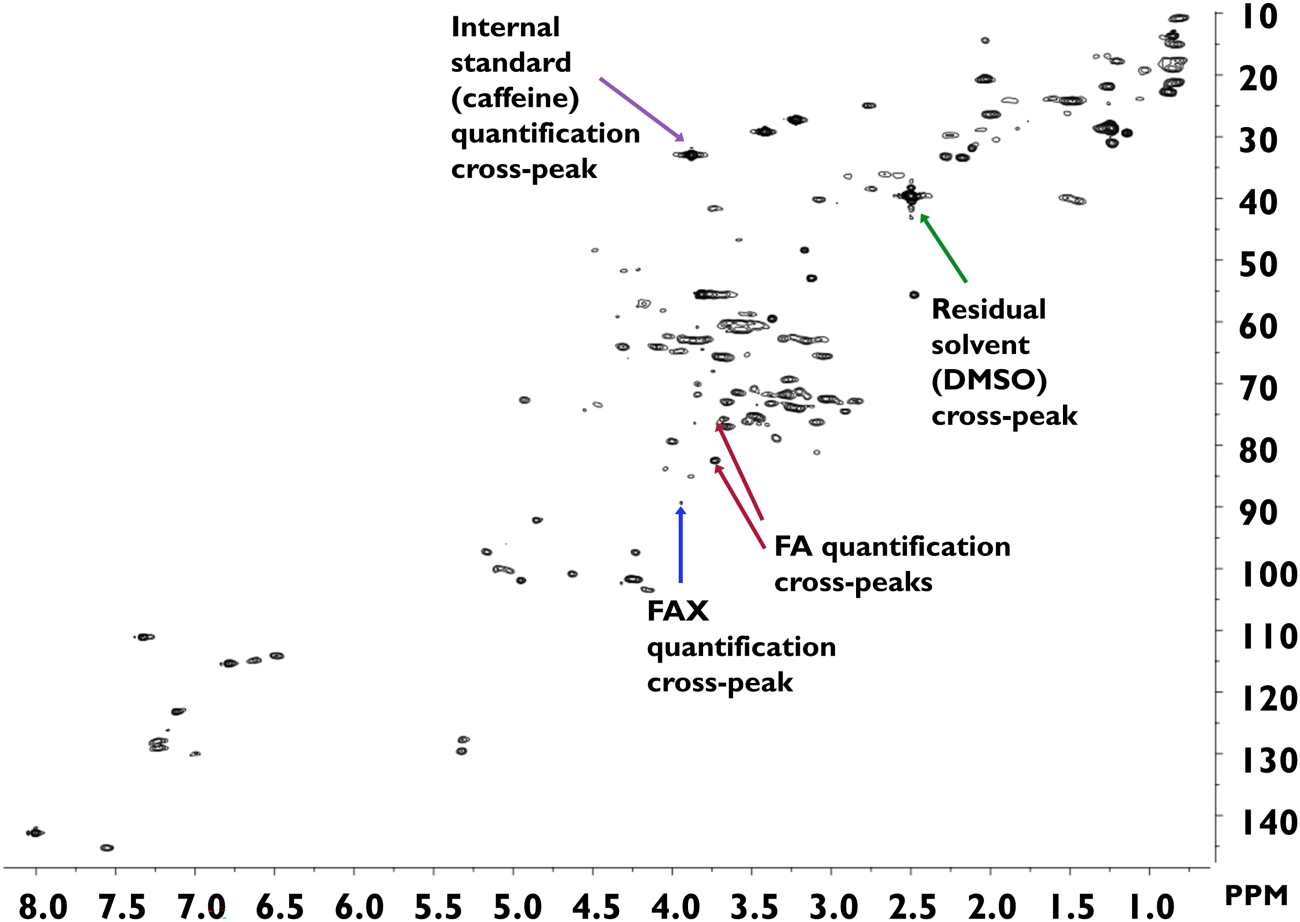
HSQC-NMR spectrum of wild rice hydrolysate. Spectra were measured in DMSO-*d*6 and calibrated against the residual DMSO signal (^1^H = 2.50 ppm; ^13^C = 39.52 ppm). DMSO, dimethyl sulfoxide; FA, 5-*O*-*trans*-feruloyl-l-arabinofuranose; FAX, β-d-xylopyranosyl-(1 → 2)-5-*O*-*trans*-feruloyl-l-arabinofuranose; FAXG, α-l-galactopyranosyl-(1 → 2)-β-d-xylopyranosyl-(1 → 2)-5-*O*-*trans*-feruloyl-l-arabinofuranose; HSQC, heteronuclear single quantum coherence spectroscopy; NMR, nuclear magnetic resonance.

The developed method was applied to survey the feruloylated side-chain profiles of popcorn, oats, wheat, and wild rice insoluble fiber (Table 3). A sample spectrum from wild rice hydrolysate is provided in Figure 3; sample spectra from popcorn, oats, and wheat are available as Supplementary Figures. FA and FAX were detected and quantified in all four cereals, but FAXG was only quantified or detected in popcorn. Table 3 provides a comparison of the percentage of ferulates captured in the NMR-based side-chain profiling method to the total monomeric ferulates released from the cereals by alkaline hydrolysis. Over 55% of the total ferulates were quantified in the NMR side-chain profiling method for oats (56%), popcorn (72%), and wheat (82%). Wild rice was an exception, with only 39% of its total ferulates being represented in the side-chain profile.

**Table 3:** Application of the semi-quantitative HSQC-NMR feruloylated side-chain profiling method to insoluble fibers from whole grains.

Grain	μmoles FA/g insoluble fiber^a^	μmoles FAX/g insoluble fiber^a^^,^^b^	μmoles FAXG/g insoluble fiber^a^	Sum of ferulates quantified in side-chain profiling method (μmol/g corrected insoluble fiber)^a^	Total monomeric ferulates as determined by alkaline hydrolysis (μmol/g corrected insoluble fiber)^a^	% of total ferulates quantified in side-chain profiling method^c^
Oats	5.39 ± 2.68	1.15 ± 0.15	ND	6.54 ± 2.58	11.81 ± 2.76	56.34 ± 21.88
Popcorn maize	75.00 ± 0.10	25.33 ± 4.35	11.18 ± 0.75^d^	111.51 ± 4.85	155.10 ± 2.35	71.92 ± 3.61
Wheat	27.39 ± 5.66	1.33 ± 0.23	ND	28.73 ± 5.89	35.66 ± 2.62	82.02 ± 24.94
Wild rice	7.21 ± 0.41	2.12 ± 0.98	ND	9.32 ± 1.33	23.89 ± 1.78	38.97 ± 3.65

a *Average ± standard deviation from triplicate determinations. All values corrected for residual protein and ash*.

b *FAXG values subtracted. Average ± standard deviation from triplicate determinations. All values corrected for residual protein and ash*.

c *[(Sum of ferulates quantified from TFA hydrolysis/total ferulates as determined by alkaline hydrolysis) × 100] ± standard deviation*.

d *Determined from diluted popcorn sample; concentration was between LOD and LOQ*.

*FA, 5-O-trans-feruloyl-l-arabinofuranose; FAX, β-d-xylopyranosyl-(1 → 2)-5-O-trans-feruloyl-l-arabinofuranose; FAXG, α-l-galactopyranosyl-(1 → 2)-β-d-xylopyranosyl-(1 → 2)-5-O-trans-feruloyl-l-arabinofuranose; HSQC, heteronuclear single quantum coherence spectroscopy; LOD, limit of detection; LOQ, limit of quantification; ND, not detected; NMR, nuclear magnetic resonance*.

**Figure 4:**
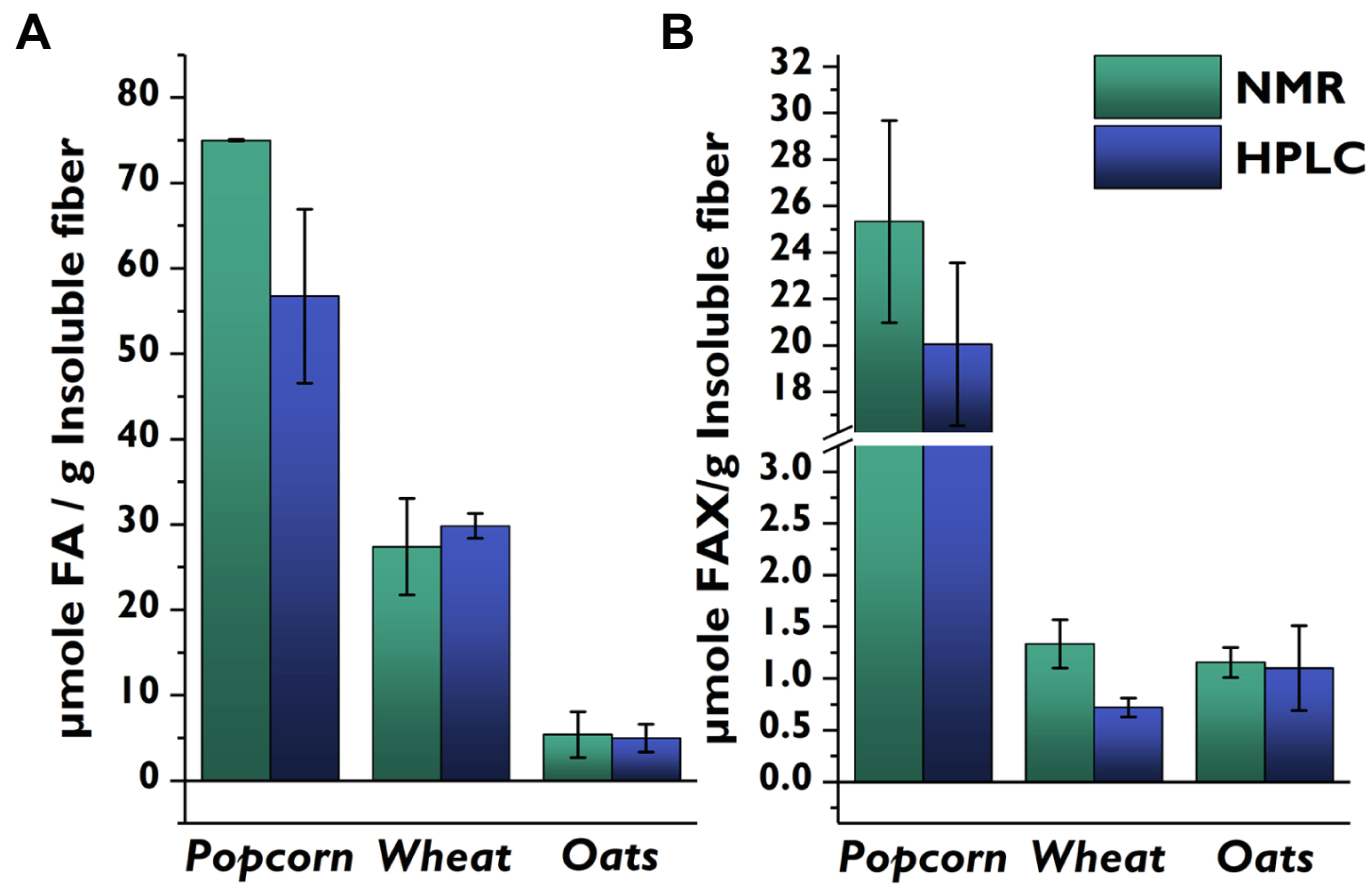
Comparison of quantities of feruloylated side-chain compounds determined from identical insoluble fiber lots of whole cereal grains using the developed HSQC-NMR method vs. a previously published HPLC-based method. **(A)**: FA; **(B)**: FAX. Values represent the average ± standard deviation from triplicate determinations. FA, 5-*O*-*trans*-feruloyl-l-arabinofuranose; FAX, β-d-xylopyranosyl-(1 → 2)-5-*O*-*trans*-feruloyl-l-arabinofuranose; HPLC, high-performance liquid chromatography; NMR, nuclear magnetic spectroscopy.

We then compared the side-chain profiles delivered by the NMR-based method to our previously developed HPLC-DAD/MS-based method (Schendel et al., 2016b) for popcorn, wheat, and oats (Figure 4). Wheat and oats had similar FA concentrations with both methods, but the FA concentration in popcorn was higher for the NMR method compared to the LC method. The higher levels produced by the NMR method likely arise from the detection of FA-containing oligosaccharides attached to a remnant of the arabinoxylan backbone. Although the mildly acidic hydrolysis conditions heavily favor cleavage of furanosidic linkages (Saulnier et al., 1995), some pyranosidic linkages are also cleaved and a small amount of feruloylated oligosaccharides still attached to a backbone remnant are also released (Schendel et al., 2015). These compounds are chromatographically separated in the LC-based method, and the FA moieties they contain are not included. In contrast, these FA moieties are quantified in the NMR-based method, and for samples rich in FA (such as popcorn), the fraction of backbone-containing oligosaccharides is large enough to be noticeable.

When comparing the FAX levels determined by the two methods, it is important to remember that popcorn was the only material which contained detectable amounts of FAXG in the NMR method, and that the FAXG value was quantified and subtracted from the FAX value. However, the more sensitive LC-based method showed that all of the samples contained FAXG, meaning that the FAX levels for oats and wheat are inflated by FAXG in the NMR method. This difference was especially apparent in the wheat samples. Additionally, it must be remembered that the FAX data from the NMR method are only semi-quantitative due to the limited linearity of the calibration curve.

### Method Limitations and Future Improvements

The NMR-based side-chain profiling method provided comparable side-chain profile data to a previously developed LC-based method. The NMR method was less sensitive than the LC-based method, and it was unable to detect the FAXG present in several cereal materials. However, FA and FAX, the two main feruloylated side-chains in cereal grains, were easily quantified in all cereal samples using the NMR-based method without having to perform the additional laboratory steps (reduction and chromatography) required by the LC-based method. Sample preparation steps (including drying) for the NMR-based method can be completed in approximately 4 h, whereas the LC-based method requires over 20 h of sample preparation time (including drying). However, measurement time for the LC-based method (<1 h) is much shorter than the NMR method (2.2 h). The sensitivity of the NMR approach could be increased by running more scans, but this would lengthen the measurement time. Non-uniform sampling is a more promising alternative for enhancing sensitivity of 2D-NMR-based methods without inflating measurement time and has been already applied for semi-quantitative profiling of carbohydrates (Fels and Bunzel, 2022). In addition, fast HSQC experiments such as ASAP-HSQC and lowCost-ASAP-HSQC support resolution in the indirect dimension and/or reducing measurement times (Fels and Bunzel, 2022, Submitted),^1^ thus being potentially valuable tools to further improve NMR-based profiling approaches for feruloylated arabinoxylans.

In conclusion, the developed method provides a solid proof of concept that 2D-NMR can be used to screen feruloylated side-chain profiles of cereal grain arabinoxylans. However, the method validation results show that the method should be approached as a semi-quantitative screening tool.

## Data Availability Statement

The raw data supporting the conclusions of this article will be made available by the authors, without undue reservation.

## Author Contributions

RS performed the experiments, analyzed the data, and drafted the manuscript. MB conceived the study idea, supervised all stages of the experimental process, and read and edited the manuscript. All authors contributed to the article and approved the submitted version.

## Funding

We acknowledge support by the KIT-Publication Fund of the Karlsruhe Institute of Technology.

## Conflict of Interest

The authors declare that the research was conducted in the absence of any commercial or financial relationships that could be construed as a potential conflict of interest.

## Publisher’s Note

All claims expressed in this article are solely those of the authors and do not necessarily represent those of their affiliated organizations, or those of the publisher, the editors and the reviewers. Any product that may be evaluated in this article, or claim that may be made by its manufacturer, is not guaranteed or endorsed by the publisher.

## Footnotes

^1^Fels, L., and Bunzel, M. (2022). HSQC-NMR based profiling approaches for raffinose family oligosaccharides in pulses. Submitted.
