# Supplementary material for: 2D-HSQC-NMR-Based Screening of Feruloylated Side-Chains of Cereal Grain Arabinoxylans
Source: Front Plant Sci. 2022 Jul 7;13:951705. doi: 10.3389/fpls.2022.951705 (PMC9301459; doi:10.3389/fpls.2022.951705)
Supplement: Supplementary file 1 [file Data_Sheet_1.pdf]

Supplemental Figures

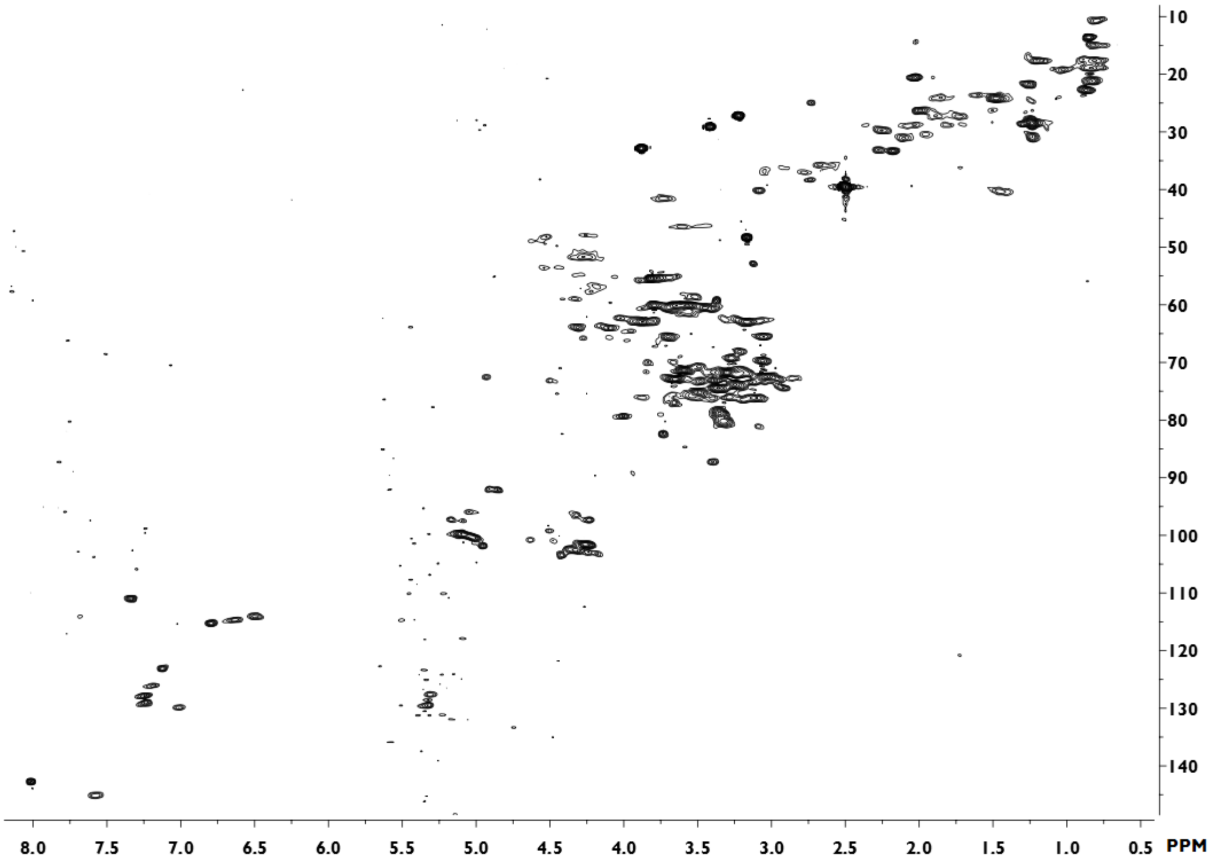

Supplemental Figure 1. HSQC-NMR spectrum of the mildly acidic hydrolysate from insoluble oat fiber. Spectrum was measured in DMSO-*d*6 and calibrated against residual DMSO signal (^{1}H = 2.50 ppm; ^{13}C = 39.52 ppm).

Abbreviations used: **HSQC:** heteronuclear single quantum coherence spectroscopy; **NMR:** nuclear magnetic resonance.

Supplemental Figures

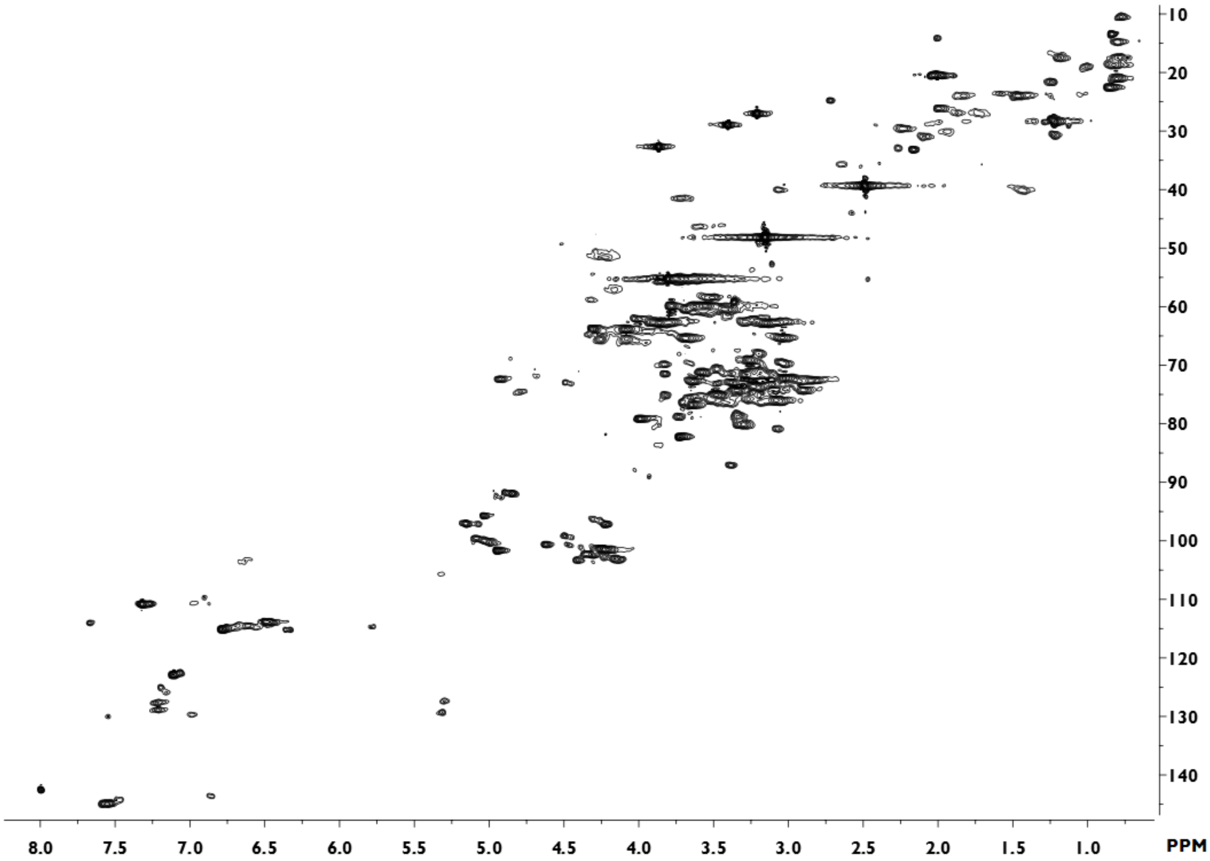

Supplemental Figure 2. HSQC-NMR spectrum of the mildly acidic hydrolysate from insoluble wheat fiber. Spectrum was measured in DMSO-*d*6 and calibrated against residual DMSO signal (^{1}H = 2.50 ppm; ^{13}C = 39.52 ppm).

Abbreviations used: **HSQC:** heteronuclear single quantum coherence spectroscopy; **NMR:** nuclear magnetic resonance.

Supplemental Figures

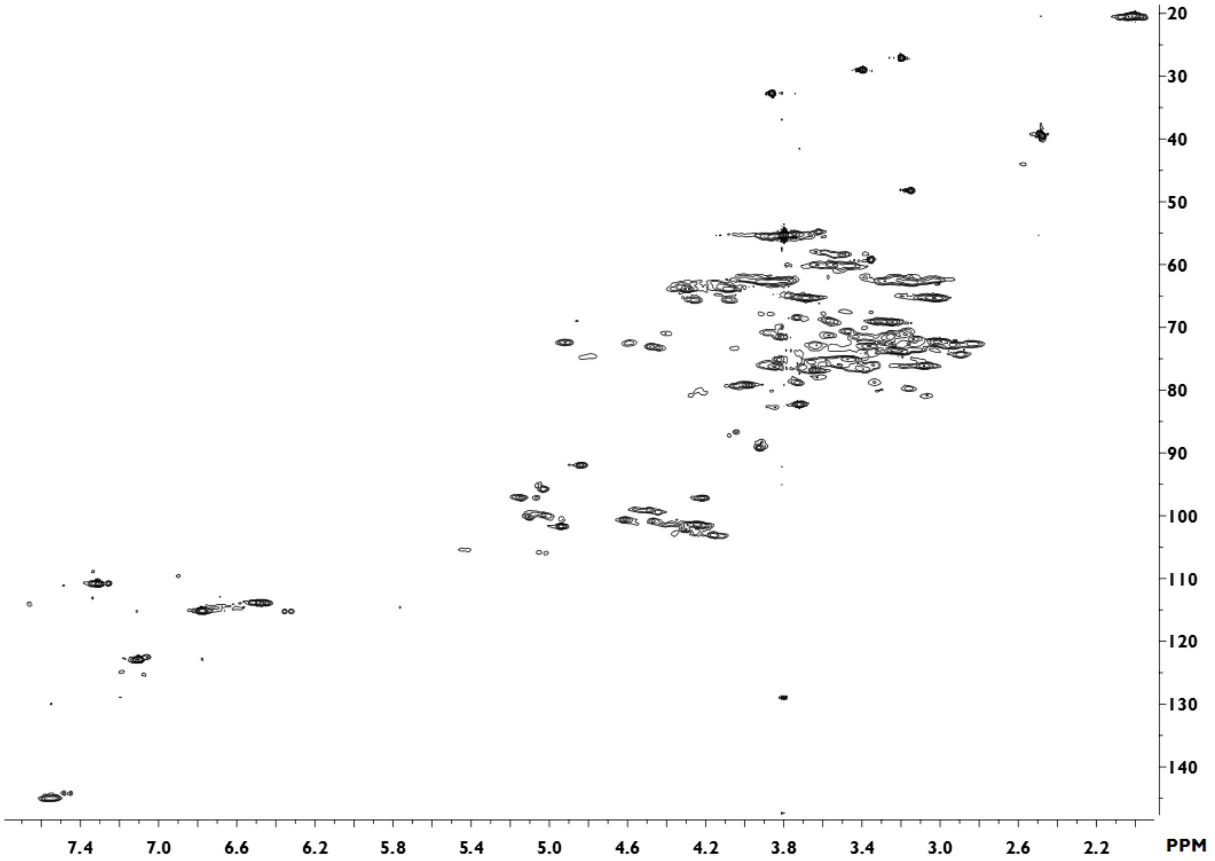

Supplemental Figure 3. HSQC-NMR spectrum of the mildly acidic hydrolysate from insoluble popcorn fiber. Spectrum was measured in DMSO-*d*6 and calibrated against residual DMSO signal (^{1}H = 2.50 ppm; ^{13}C = 39.52 ppm).

Abbreviations used: **HSQC:** heteronuclear single quantum coherence spectroscopy; **NMR:** nuclear magnetic resonance.
